# The clinical importance of air plethysmography in the assessment of chronic venous disease

**DOI:** 10.1590/1677-5449.002116

**Published:** 2016

**Authors:** Nei Rodrigues Alves Dezotti, Marcelo Bellini Dalio, Maurício Serra Ribeiro, Carlos Eli Piccinato, Edwaldo Edner Joviliano

**Affiliations:** 1 Universidade de São Paulo – USP, Faculdade de Medicina de Ribeirão Preto, Departamento de Cirurgia e Anatomia, Divisão de Cirurgia Vascular e Endovascular, Ribeirão Preto, SP, Brazil.

**Keywords:** air plethysmography, chronic venous disease, varicose veins, venous thrombosis, leg ulcer, pletismografia a ar, doença venosa crônica, varizes, trombose venosa, úlcera de perna

## Abstract

Air plethysmography is a non-invasive test that can quantify venous reflux and obstruction by measuring volume changes in the leg. Its findings correlate with clinical and hemodynamic measures. It can quantitatively assess several components of venous hemodynamics: valvular reflux, calf muscle pump function, and venous obstruction. Although clinical uses of air plethysmography have been validated, it is used almost exclusively for medical research. Air plethysmography can be used to assess chronic venous disease, to evaluate improvement after venous surgery, to diagnose acute and past episodes of deep venous thrombosis, to evaluate compression stocking therapy, to study the physiological implications of high-heeled shoes in healthy women, and even to evaluate the probability of ulcer healing.

## Resumo

A pletismografia a ar é um método não invasivo que pode quantificar refluxo e obstrução venosa medindo alterações no volume das pernas. Seus achados se correlacionam com parâmetros clínicos e hemodinâmicos. Ela pode fornecer informações quantitativas dos diferentes componentes da hemodinâmica venosa: refluxo valvular, função de bomba muscular da panturrilha e obstrução venosa. Apesar de ter seu uso clínico validado, a pletismografia a ar é usada quase que exclusivamente para pesquisa. Ela pode ser usada para avaliar a doença venosa crônica, mensurar o ganho hemodinâmico após cirurgia venosa, diagnosticar trombose venosa profunda atual ou prévia, avaliar os efeitos da elastocompressão, estudar as implicações fisiológicas do uso de salto alto em mulheres e também avaliar a probabilidade de cura de uma úlcera venosa.

## INTRODUCTION

Chronic venous disease (CVD) includes a spectrum of clinical presentations ranging from uncomplicated telangiectasis and varicose veins to venous ulceration.^1^ It represents an important public health problem with economic and social consequences.^2^^-^ 5 Prevalence is about 20 to 73% in females and 15 to 56% in males.^6^ The combination of skin abnormalities and sustained venous hypertension is referred to as chronic venous insufficiency (CVI). Manifestations of CVD are the result of outflow reflux, obstruction, or a combination of both. These processes may be primary or secondary to other conditions, such as deep venous thrombosis (DVT).^1^

Duplex scanning and venography can provide the anatomic and physiologic information necessary to diagnose and treat venous reflux and/or obstruction in the superficial, deep, and perforator systems.^7^ Air plethysmography (APG) has been introduced as an additional tool for the evaluation of venous hemodynamics.^8^^,^ 9 APG is a non-invasive test that can quantify venous reflux and obstruction by measuring volume changes in the leg.^10^ Its findings correlate with clinical and hemodynamic measures.^11^

Although clinical uses of APG have been validated, it is almost exclusively used for medical research. The purpose of this review is to discuss the applications of APG for clinical assessment of chronic venous disease.

## STANDARD AIR PLETHYSMOGRAPHY TECHNIQUE

**Figure 1:**
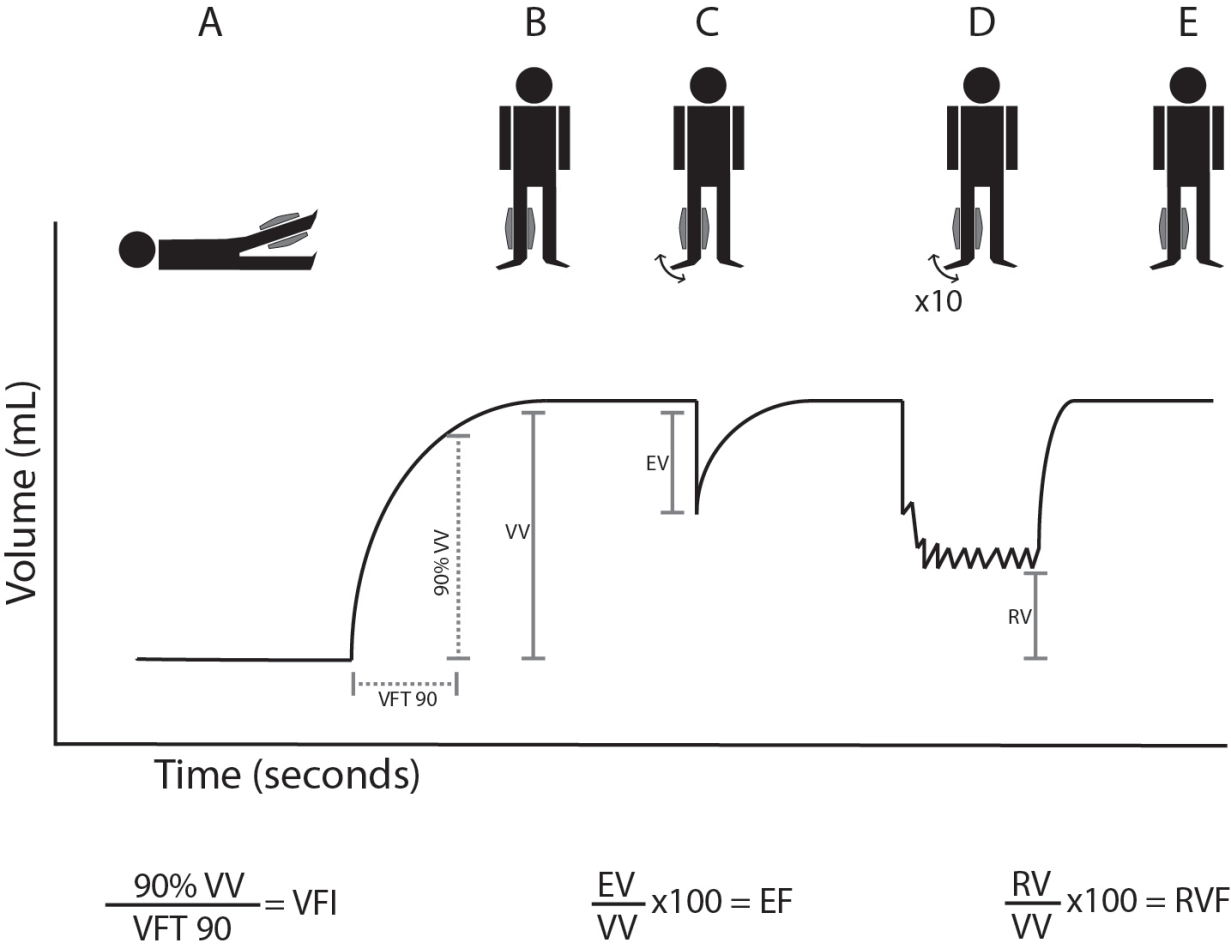
Air plethysmography data obtained from tracings. EF: ejection fraction; EV: ejected volume; RV: residual volume; RVF: residual volume fraction; VFI: venous filling index; VFT: venous filling time; VV: venous volume. (A) Baseline with leg elevated 45 degrees; (B) The patient stands and a plateau in leg volume is reached; (C) The patient is then asked to perform a single heel-raise maneuver and a decrease in volume is recorded; (D) The patient is asked to perform 10 heel-raise maneuvers and a new plateau is recorded; (E) Finally, the patient is asked to remain standing.

Christopoulos et al.^12^ have described validation and the reproducibility and results of APG in detail, both in normal volunteers and in patients with superficial or deep venous disease. In order to evaluate venous reflux, APG is performed with a 35 cm-long polyvinyl chloride air chamber (5 L capacity) that surrounds the leg from knee to ankle and is connected to a pressure transducer and chart recorder. The pressure transducer is calibrated with 100 mL of air after the air chamber is fitted around the leg, with the patient supine. The leg is elevated to 45 degrees to empty the veins and a baseline reading is obtained (Figure 1A). The patient is then asked to stand, putting body weight on the opposite leg. The increase in leg volume is observed until a plateau is reached, indicating that the veins are full (Figure 1B). This plateau corresponds to the functional venous volume (VV). The time taken to achieve 90 percent of venous volume has been defined as venous filling time 90 (VFT 90). The venous filling index (VFI) is calculated by dividing 90% of VV by VFT90. The patient is then asked to perform a single heel-raise maneuver. The resultant decrease recorded is the ejected volume (EV), caused by contraction of the calf muscle (Figure 1C). The ejection fraction (EF) is calculated by dividing the ejection volume by the venous volume and multiplying by 100. After a new plateau is reached, 10 heel-raise maneuvers are performed to reach another plateau, representing residual volume (Figure 1D). The residual volume (RV) is the volume at the end of exercise. Finally, the patient is asked to remain standing (Figure 1E). The residual volume fraction (RVF) is calculated by dividing the RV by the VV and multiplying by 100. The VFI is an index of global venous reflux, the EF is a reflection of calf muscle pump function, and the RVF is a reflection of ambulatory venous pressure.^8^^,^ 9

In order to evaluate venous obstruction, APG is performed using an 11 cm pneumatic cuff with a 40 cm long bladder placed around the proximal thigh to act as a partially occluding tourniquet. After calibration, with the patient in supine position, the tourniquet is inflated to 70 mmHg and maintained until a maximal venous volume is reached and the chart recorder traces a plateau. Upon rapid deflation of the tourniquet, venous outflow is recorded. The outflow fraction (OF) is obtained by dividing the amount of venous volume emptied in 1 second by the venous volume and multiplying by 100. OF values lower than 38% suggest venous obstruction.^11^

## APPLICATIONS OF AIR PLETHYSMOGRAPHY IN THE CLINICAL ASSESSMENT OF CHRONIC VENOUS DISEASE

Some important applications of APG in evaluation of lower extremity chronic venous disease and venous function are discussed below:

### Evaluation of hemodynamics and severity of chronic venous disease

The parameters VFI, EF, RVF and OF obtained using APG provide a quantitative evaluation of severity of chronic venous insufficiency.^13^ VFI provides an overall assessment of the degree of venous valve insufficiency. EF is the percentage of VV that is expelled from the calf by one heel-raise maneuver and assesses the efficiency of the calf muscle pump. RVF represents the percent of the VV remaining after 10 heel-raise maneuvers. This has been shown to correlate with ambulatory venous pressure.

In the past, ambulatory venous pressure was considered the gold standard for studying hemodynamic changes in venous disease. It was also used for validating the results of other non-invasive tests and for predicting the results of venous surgery. However, it is an invasive procedure and therefore cannot be used repeatedly or as a screening test. Recently, non-invasive tests have become available for anatomic and functional study of CVD. A number of studies have quantified venous reflux by APG and showed that APG parameters correlate with clinical stages. Christopoulos et al.^14^ found that VFI, which measures the degree of venous reflux, corresponds with worsening clinical classes. Engelhorn et al.^15^ demonstrated that APG can determine the severity of CVD and that VFI is the parameter which best correlates with clinical severity. The role of VFI in assessing the clinical severity of CVD has also been demonstrated by Oliveira et al.^16^ Similarly, deterioration has been reported in all parameters measured by APG as clinical stages progressed.^17^

In addition to assessment of reflux, APG can also be used to evaluate calf pump function. EF is a reflection of calf muscle pump function as well as of outflow resistance, particularly in post-thrombotic limbs with destroyed venous sinuses or recanalized deep veins.^18^ Furthermore, RVF is the net result of valvular reflux and calf muscle pump function. RVF correlates with ambulatory venous pressure.^19^

Qualitative assessment of the significance of superficial and deep venous reflux in different clinical classes can also be conducted using APG. Christopoulos et al.^20^ managed to measure the amount that the superficial vein and the deep vein each contributed to reflux separately by using a tourniquet placed at the knee level to occlude the superficial vein. They found that it was the magnitude of reflux rather than its location that was responsible for the sequelae of venous disease.

### Evaluation of changes to venous hemodynamics after surgical treatment of chronic venous disease

APG can be used to evaluate outcomes after surgical treatment of CVD. Many investigations have used APG to analyze improvements in venous hemodynamics after primary varicose vein surgery.^21^^-^ 25 Generally, varicose vein surgery promotes a reduction in VFI and an increase in EF in the operated lower limb. These findings are maintained 5 years after surgery.^26^ Superficial varicose vein stripping contributes to relief of venous stasis and prevents pathophysiological evolution of chronic venous disease irrespective of clinical severity.

APG can also be used to evaluate the correlation between venous hemodynamic gain and CEAP classification^27^ after surgical treatment of primary varicose veins. Surgical treatment offers benefits for all classes, but the greatest hemodynamic gains are observed in the subset with higher clinical severity (C5 and C6).^28^

APG can also be used to evaluate the outcomes of deep venous surgery. Sakuda et al.^29^ described improved hemodynamic parameters in patients with CVD with deep venous reflux who had undergone external valvoplasty.

### Diagnosis and follow-up of deep venous thrombosis

In most major medical centers, venous duplex scanning is the procedure of choice for initial evaluation of patients with DVT.^30^ However, APG can be used to evaluate venous obstruction and can diagnose both acute DVT and a past episode.^31^^,^ 32 Using APG to measure OF may be a practical alternative for screening symptomatic patients and also for detecting hemodynamic changes after anticoagulation treatment.

Patients who develop chronic deep venous obstruction after a DVT episode have more severe hemodynamic dysfunction than those without obstruction.^33^ Therefore, objective assessment of venous obstruction is important to clarification of the pathophysiology of patients with CVD. Since APG can identify and quantify venous obstruction, it may be of great value in follow-up of DVT patients.

However, a recent study failed to demonstrate that OF, EF, and RVF obtained by APG are reliable for diagnosis of chronic deep venous obstruction.^34^ The authors stated that collateralization and recanalization over time can influence the outflow capacity and may therefore cause false negative results. They also consider that APG may nevertheless still be a good physiologic tool for gaining greater insight into the hemodynamics of venous disease, but that its use for diagnosing chronic deep venous obstruction is unwarranted in daily clinical practice.

### Evaluation of effectiveness of compression stocking therapy

The degree of venous reflux and improvement in calf pump function in patients who have been prescribed compression stockings can be tested serially.^35^ The patient’s reflux and calf ejection fraction can be plotted to show the effect of compression on venous hemodynamics. This may identify some patients who are not benefiting from compression stockings.

### Evaluation of the physiological implications of high-heeled shoes on venous return for healthy women

Walking in high-heeled shoes is a common cause of venous complains such as pain, fatigue, and feelings of heavy legs. A recent investigation used APG to test whether women wearing a range of high-heeled shoes exhibited impaired venous return when compared with themselves walking barefoot.^36^ The results indicate that high heels reduce muscle pump function, as demonstrated by reduced EF and increased RVF values. Continuous use of high heels tends to cause venous hypertension in the lower limbs and may represent a causal factor of venous disease symptoms.

### Evaluation of the probability of ulcer healing

Venous leg ulcers are larger, long-standing, and difficult to heal when associated with calf muscle failure. Impaired muscle pump function can be revealed by APG and could be a prognostic factor for delayed healing of leg ulcers. Ulcer patients with poor prognosis according to plethysmographic findings who do not recovery quickly after standard management should be considered for advanced therapies.^37^

Other potential situations in which APG may have a role in defining venous hemodynamic changes are: pregnant women, athletes, obese people, and for differential diagnosis of lower limb edema. These situations are being studied in ongoing investigations.

## DIFFICULTIES WITH USING AIR PLETHYSMOGRAPHY IN CLINICAL SETTINGS

Although it is a noninvasive and relatively inexpensive method, APG can be technically difficult. It is highly dependent on accurate calibration and because of this it can be considered an examiner-dependent test. Minimal technical errors during measurements invalidate the test and make it obligatory to repeat the test from the start. Calibration and frequent restarting mean that APG is often a time-consuming procedure. Obesity can also influence results, making APG parameters inappropriate.

As stated above, APG is currently almost exclusively used for medical research. Despite its low cost and non-invasive nature, APG is rarely found in clinical vascular laboratories. In Brazil, this fact could be partly explained by the absence of a domestic APG device manufacturer. Until recently, there was an APG device produced in Brazil that was available at a reasonable cost. Nowadays, all APG devices in Brazil must be imported, which makes costs considerably higher.

## CONCLUSION

Air plethysmography is a non-invasive test that can be used to quantitatively assess several different components of venous hemodynamics: valvular reflux, calf muscle pump function, and venous obstruction. It can be used to assess chronic venous disease, to evaluate improvement after venous surgery, to diagnose acute and past episodes of deep venous thrombosis, to evaluate compression stocking therapy, to study the physiological implications of high-heeled shoes in healthy women, and even to evaluate the probability of ulcer healing.
